# Supplementary material for: Model design for non-parametric phylodynamic inference and applications to pathogen surveillance
Source: bioRxiv. 2021 Aug 16:2021.01.18.427056. Preprint. [Version 2] doi: 10.1101/2021.01.18.427056 (PMC8382123; doi:10.1101/2021.01.18.427056)
Supplement: 1 [file NIHPP2021.01.18.427056V2-supplement-1.pdf]

bioRxiv preprint doi: https://doi.org/10.1101/2021.01.18.427056; this version posted August 16, 2021. The copyright holder for this preprint (which was not certified by peer review) is the author/funder, who has granted bioRxiv a license to display the preprint in perpetuity. It is made available under aCC-BY 4.0 International license.

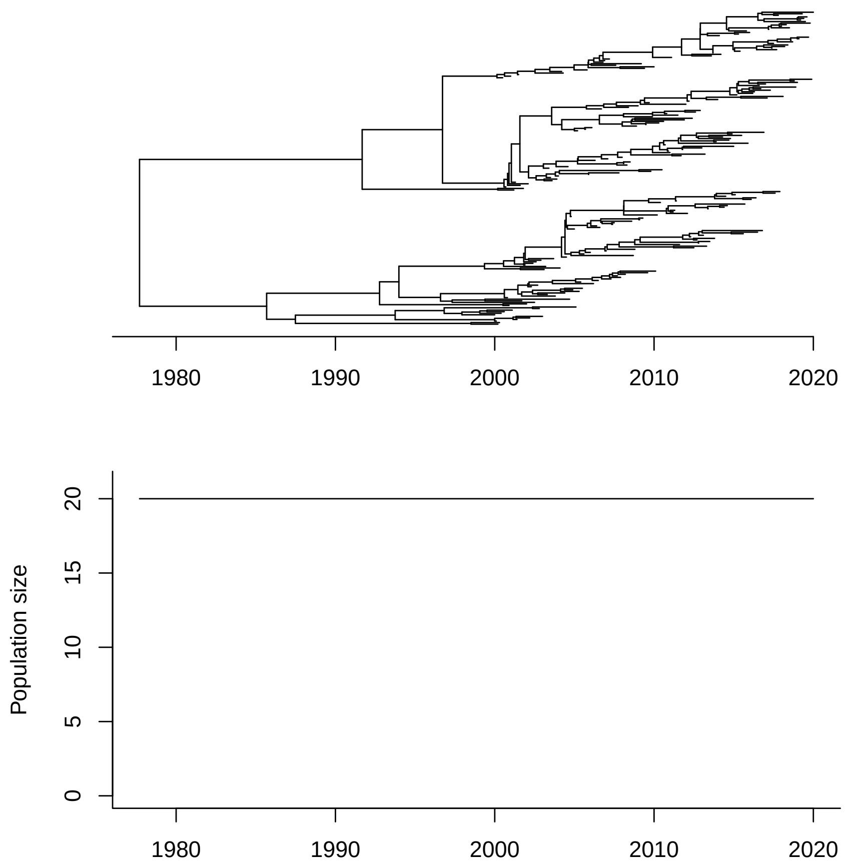

Figure S1: Simulated phylogeny using a constant demographic function.

bioRxiv preprint doi: https://doi.org/10.1101/2021.01.18.427056; this version posted August 16, 2021. The copyright holder for this preprint (which was not certified by peer review) is the author/funder, who has granted bioRxiv a license to display the preprint in perpetuity. It is made available under aCC-BY 4.0 International license.

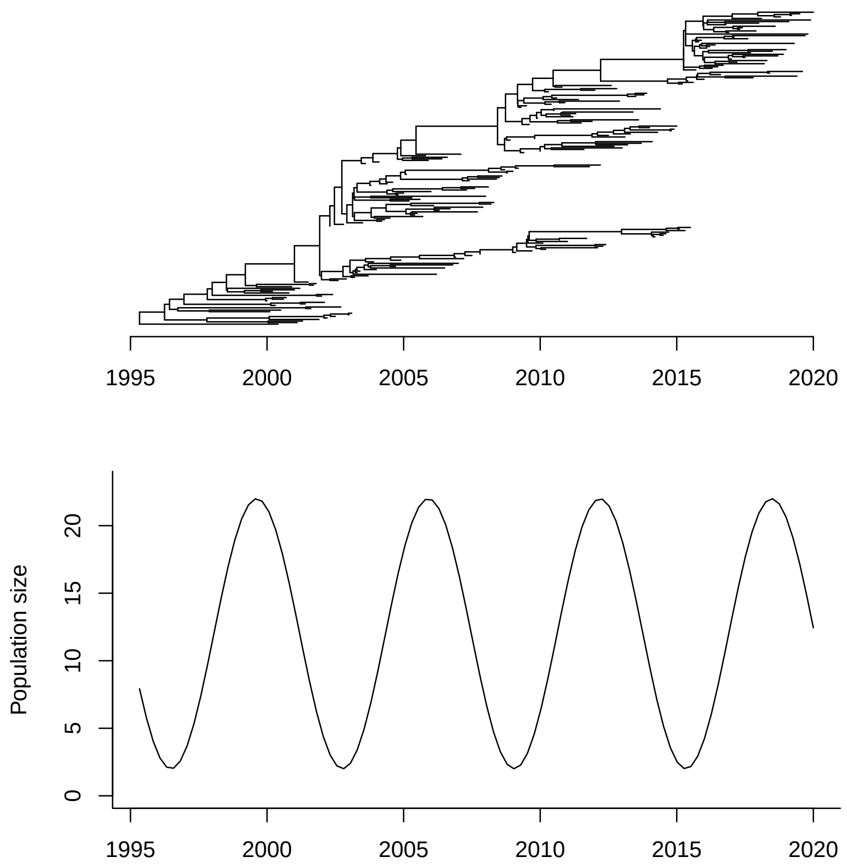

Figure S2: Simulated phylogeny using a sinusoidal demographic function.

bioRxiv preprint doi: https://doi.org/10.1101/2021.01.18.427056; this version posted August 16, 2021. The copyright holder for this preprint (which was not certified by peer review) is the author/funder, who has granted bioRxiv a license to display the preprint in perpetuity. It is made available under aCC-BY 4.0 International license.

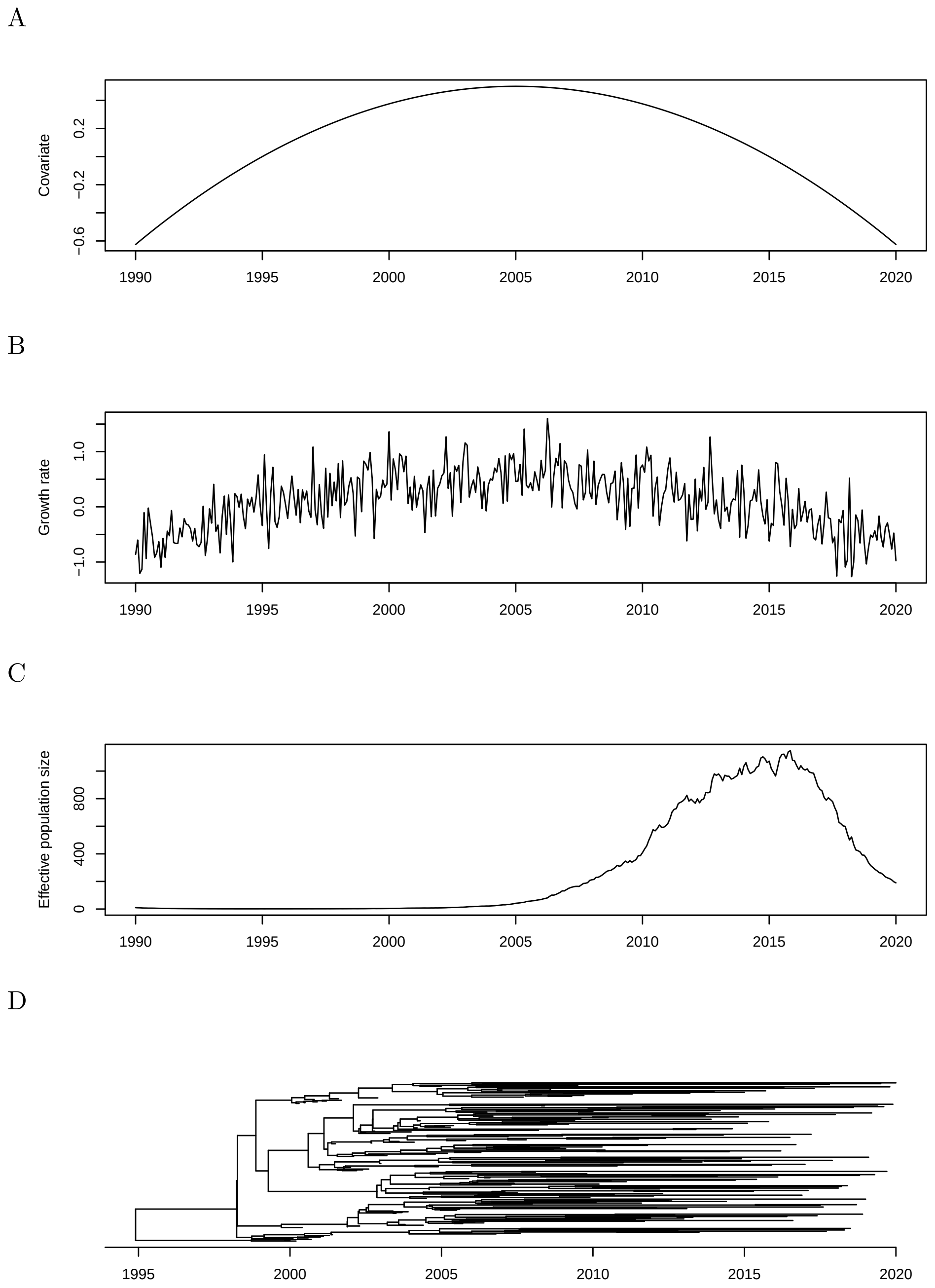

Figure S3: Example of simulation with covariate data driving the growth rate. (A) Covariate data following a quadratic function. (B) Growth rate equal to the covariate data plus some Gaussian noise. (C) Effective population size. (D) Dated phylogeny.

bioRxiv preprint doi: https://doi.org/10.1101/2021.01.18.427056; this version posted August 16, 2021. The copyright holder for this preprint (which was not certified by peer review) is the author/funder, who has granted bioRxiv a license to display the preprint in perpetuity. It is made available under aCC-BY 4.0 International license.

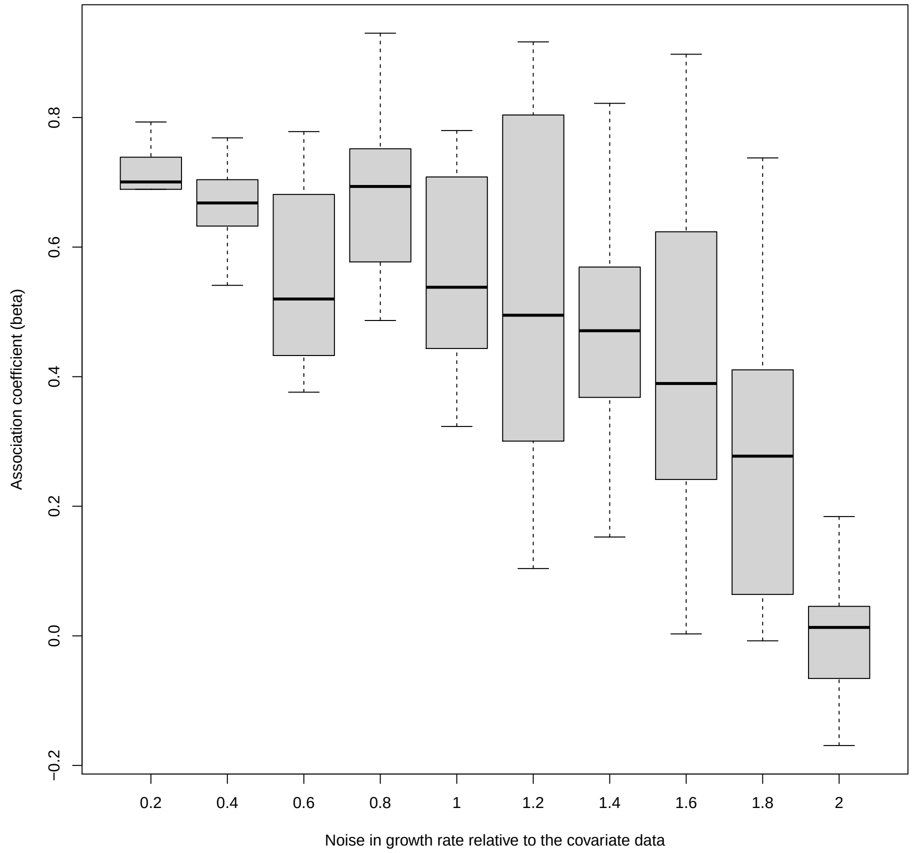

Figure S4: Results of the covariate analysis. For each value of the Gaussian noise (x-axis) ten simulations were performed and the inferred values of the association coefficient β are shown (y-axis) as boxplots.
